# Genome-wide analysis of the DREB family genes and functional identification of the involvement of *BrDREB2B* in abiotic stress in wucai (*Brassica campestris* L.)

**DOI:** 10.1186/s12864-022-08812-1

**Published:** 2022-08-17

**Authors:** Ying Wu, Liting Zhang, Libing Nie, Yushan Zheng, Shidong Zhu, Jinfeng Hou, Renjie Li, Guohu Chen, Xiaoyan Tang, Chenggang Wang, Lingyun Yuan

**Affiliations:** 1 College of Horticulture, Anhui Agricultural University, 230036 Hefei, Anhui China; 2 College of Horticulture and Forestry, Huazhong Agricultural University, 430070 Wuhan, Hubei China; 3 College of Horticulture, Nanjing Agricultural University, 210095 Nanjing, Jiangsu China; 4 Wanjiang Vegetable Industrial Technology Institute, 238200 Maanshan, Anhui China

**Keywords:** *Brassica campestris*, *DREB* gene family, Genome-wide identification, Functional verification

## Abstract

Dehydration responsive element binding protein (DREB) is a significant transcription factor class known to be implicated in abiotic stresses. In this study, we systematically conducted a genome-wide identification and expression analysis of the *DREB* gene family, including gene structures, evolutionary relationships, chromosome distribution, conserved domains, and expression patterns. A total of 65 *DREB* family gene members were identified in Chinese cabbage (*Brassica rapa* L.) and were classified into five subgroups based on phylogenetic analysis. Through analysis of the conserved domains of *BrDREB* family genes, only one exon existed in the gene structure. Through the analysis of *cis*-acting elements, these genes were mainly involved in hormone regulation and adversity stress. In order to identify the function of *BrDREB2B*, overexpressed transgenic *Arabidopsis* was constructed. After different stress treatments, the germination rate, root growth, survival rate, and various plant physiological indicators were measured. The results showed that transgenic *Arabidopsis thaliana* plants overexpressing *BrDREB2B* exhibited enhanced tolerance to salt, heat and drought stresses. Taken together, our results are the first to report the *BrDREB2B* gene response to drought and heat stresses in Chinese cabbage and provide a basis for further studies to determine the function of *BrDREBs* in response to abiotic stresses.

**Supplementary Information:**

The online version contains supplementary material available at 10.1186/s12864-022-08812-1.

## Introduction

Abiotic stresses, such as high and low temperatures, drought, and salinization, have severe adverse effects on plant growth and development [1, 2]. At the transcription level, transcription factors (TFs) can specifically bind to *cis*-acting elements in the promoter region, thereby regulating the expression of plant stress response genes. Therefore, the key role of transcription factors in plants under environmental stress has attracted increasing research attention [3, 4].

The AP2/ERF (APETALA2/ethylene-responsive factor) superfamily, which is one of the largest groups of TFs families, is involved in the regulation of plant developmental processes and biotic and abiotic stress responses [5, 6]. The genes of the superfamily are characterized by encoding at least one APETALA2 (AP2) domain and can be further separated into ERF, AP2, DREB, RAV and Soloist families. The ERF family contains a single AP2 domain, while the AP2 gene family contains one or two AP2 domains [7, 8]. The RAV (Related to ABI3 and VP1) family encodes proteins encoding a single AP2 domain plus an additional B3 domain [9]. There is another subfamily with a single AP2 domain called soloist [8].

DREB transcription factors are a subgroup of the AP2/ERF (APETALA2/ethylene-responsive factor) gene family, which contain a single conserved AP2 domain that specifically interacts with the dehydration responsive element (DRE) (core motif A/GCCGAC) [10–12]. DREB transcription factors bind to the DRE and the C-repeat responsive (CRT) *cis*-element in the promoter region of stress resistance genes to regulate the expression of a series of downstream genes and enhance the resistance of plants to different abiotic stresses [13]. Recently, a number of *DREB* homologous genes have been identified from different plants, such as *Arabidopsis* [13, 14], rice [15], wheat [16], and soybean [17].

Based on the sequence identities of the DNA-binding domains, DREB proteins can be divided into six subgroups (A1-A6) [18]. *DREB* members in different subgroups play different roles in plants. In subgroup A2, *DREB2A* and *DREB2B*, as the main functional genes in *DREB2*s, have been reported to play an important role in resistance to dehydration, high salt, and heat shock [19]. In *Arabidopsis*, *DREB2A* and *DREB2B* were identified and actively responded to dehydration and high salinity stress [20]. In mung bean (*Vigna radiata* L.), *VrDREB2A* was induced by drought and salt stresses, and heterologous expression of *VrDREB2A* enhanced the tolerance to drought and high salt stress without growth retardation in *Arabidopsis* [21]. In mustard (*Brassica juncea* L), *BjDREB2* playing a pivotal role in ABA-independent gene expression under drought stress [22].

Wucai (*Brassica campestris* L. ssp. *chinensis* var. *rosularis*), a variant of non-heading Chinese cabbage, is a semi-hardy vegetable widely grown in the Yangtze-Huai River basin [23, 24]. Recently, abiotic stresses such as high temperature, freezing, and drought have greatly affected the yield and quality of wucai. Therefore, improvement of resistance to abiotic stress in wucai is desirable. There are few studies on the molecular mechanism of stress resistance, the expression regulation of genes related to stress resistance, and the stress signal transduction mechanisms. In this study, the genome-wide identification and mining of DREB transcription factor family genes in Chinese cabbage were systematically performed. We also determined the gene structure, evolution relationships, *cis-*acting element evolution relationship, and chromosome distribution of the Chinese cabbage *DREB* gene family in detail. In addition, we cloned and characterized the *BrDREB2B* gene in wucai. Furthermore, the overexpression of *BrDREB2B* enhanced the drought, high-salt, and heat tolerance of transgenic *Arabidopsis* plants. Our study provides a theoretical foundation to understand the role of *BrDREBs* in resistance to abiotic stress and a basis for further study of the biological functions of *DREB* genes.

## Materials and methods

### Genome-wide identification of *BrDREB* genes in chinese cabbage

The Hidden Markov Model (HMM) profile of the AP2 domain (PF00847) was downloaded from the protein family database (http://pfam.xfam.org/) with an E-value < 0.001, and was used to search the Chinese cabbage genome database (http://brassicadb.org/brad/) [25–28]. There is only one AP2 domain in the DREB subfamily member protein, and the 14th and 19th positions are valine and glutamic acid, respectively. The NCBI-CDD database (https://www.ncbi.nlm.nih.gov/Structure/bwrpsb/bwrpsb.cgi) and SMART website (http://smart.embl-heidelberg.de/) were used for domain prediction and identified members of the DREB family of Chinese cabbage. The physical and chemical properties of the DREB family gene were analysed using the Expasy website (https://web.expasy.org/compute_pi/) [29], and the subcellular locations of DREB family members were predicted using the WoLF PSORT website (https://wolfpsort.hgc.jp) [30].

### Conserved motifs and cluster analysis of the *BrDREB* gene family

We predicted the conserved motifs of the BrDREB protein using the MEME website (http://meme-suite.org/tools/meme). The identified Chinese cabbage DREB amino acid sequence and the reported *Arabidopsis* DREB amino acid sequence were aligned using MEGA7.0 software [31], and unrooted phylogenetic trees were constructed by the neighbor-joining (NJ) method. We used the bootstrap method in the phylogeny test, and the number of bootstrap replications was set to 1000.

### Analysis of *cis*-acting elements of the *BrDREB* gene family

The sequence upstream (2000 bp) of the transcriptional start site was used to identify *cis*-elements in the promoter sequences of the *DREB* family genes in *B*. *rapa*. The *cis*-acting elements were predicted and analyzed using the PlantCARE website (http://bioinformatics.psb.ugent.be/webtools/plantcare/htmL/) [32] and were visualized with TBtools software [33].

### Chromosome distribution and collinearity analysis

The chromosome distribution of the *BrDREB* genes were extracted from the *B. rapa* genome annotation gff3 file for collinearity analysis by TBtools software. Analyses of interspecies synteny between *BrDREBs* and *AtDREBs* were investigated using MCScan X [34].

### Plant materials and stress treatments

Wucai (*Brassica campestris* L.) cultivar WS-1, a high-generation inbred line, was selected from Anhui Agricultural University. Seedlings were germinated and cultured in a growth chamber at 24 ± 1 °C (day) and 16 ± 1 °C (night), with a relative humidity of 75–80% and a light intensity of 300 µmol·m^− 2^·s^− 1^ under a 14-h/10-h (light/dark) photoperiod. After 20 days, unfolded functional leaves were collected for RNA extraction by total RNA kit (Takara Biomedical Technology Co., Beijing, China). Primer software v6.0 (Premier Biosoft International, Palo Alto, CA, USA) was used to design specific gene primers (Table S3). The gene encoding actin was used as the control. RT-qPCR was performed using SYBR GREEN Master Mix (Vazyme Biotechnology Co., Ltd., Nanjing, China). Relative gene expression levels were calculated using the 2^−ΔΔCT^ method [35].

*Arabidopsis* (*Columbia-0*) plants were placed in a growth chamber at 25 °C (light for 18 h) and 15 °C (dark for 6 h), with a light intensity of 300 µmol·m^− 2^·s^− 1^. After 30 days, leaves of wild type and transgenic *Arabidopsis* were collected for analysis at the gene expression level. Transgenic T_3_ generation and wild type seedings were treated with 1/2 Murashige and Skoog (MS) liquid medium containing 150 mM NaCl [36] and 250 mmol·L^− 1^ mannito [37], respectively. Seedings in 1/2 MS liquid medium were subjected to 40 °C to mimic heat stress [38]. Seedlings under normal conditions were used as the control.

### Generation of *BrDREB2B*‑overexpressing *Arabidopsis*

*BrDREB2B* cDNA was cloned into the pMD19-T simple vector. *BrDREB2B* was inserted into plasmid pCambia1305-35 S-nFLAG-cMYC, and transgenic *Arabidopsis* plants were cultivated by the inflorescence infection method. The regenerated plants were placed in a medium containing 50% hygromycin B and grown in an incubator. Four weeks later, the copy number of *BrDREB2B* of each transgenic line was detected by Quantitative Real-time PCR (qRT-PCR), and three overexpression positive transgenic lines were identified (OE#1, OE#7, and OE#8).

### Physiological index measurement of transgenic plants

The transgenic T_3_ generation seeds and wild-type seeds were planted on ½ MS medium at 40 °C, ½ MS medium with 150 mmol·L^− 1^ NaCl, and ½ MS medium with 250 mmol·L^− 1^ mannitol. Normal ½ MS medium was used as the control group, and the germination rate was observed after one week. The 5-day-old transgenic and wild-type seedlings were carefully transferred to ½ MS medium containing 150 mmol·L^− 1^ NaCl. Normal ½ MS medium was used as the control group. After 2 weeks, 20 transgenic and wild-type plants were randomly selected for root length measurement. The 3-week-old transgenic and wild-type plants were placed in an artificial climate incubator at 40 °C/30°C (day for 18 h/night for 6 h) for 3 d, compared with 25 °C/15°C (day for 18 h/night for 6 h) as a control. The growth of transgenic and wild-type plants was observed. Transgenic and wild-type plants were deprived of water for 14 d and then re-watered for 3 d. We observed the growth of the plants and determined the survival rate.

Relative electrical conductivity (REC) was measured according to the method described by Baziramakenga et al. with some modifications [39]. The H_2_O_2_ and O_2_^−^ content, as well as the total antioxidant capacity, were measured using the Solarbio reagent kit (Cat #BC3595, 1290 and 1315, Beijing Solarbio Science & Technology Co., Ltd., Beijing, China).

## Results

### Identification and characterization of the *BrDREBs*

A total of 65 *BrDREB* genes were identified from the *Brassica rapa* genome (Table S1), which are unevenly distributed on 10 chromosomes. The ORFs of the BrDREB genes ranged from 471 to 1158 bp in length, encoding polypeptides 156 aa to 385 aa in length. The predicted molecular mass was between 17.46 and 43.05 kDa, and the isoelectric point also varied widely from 4.48 to 9.73, suggesting that different DREB proteins might operate in different microenvironments. Predictions from the WoLF PSORT website showed that seven genes were subcellularly located on the chloroplast, and the other 58 genes were all located on the nucleus.

### Gene structure and conserved motif analysis of the *BrDREB* gene family

**Fig. 1:**
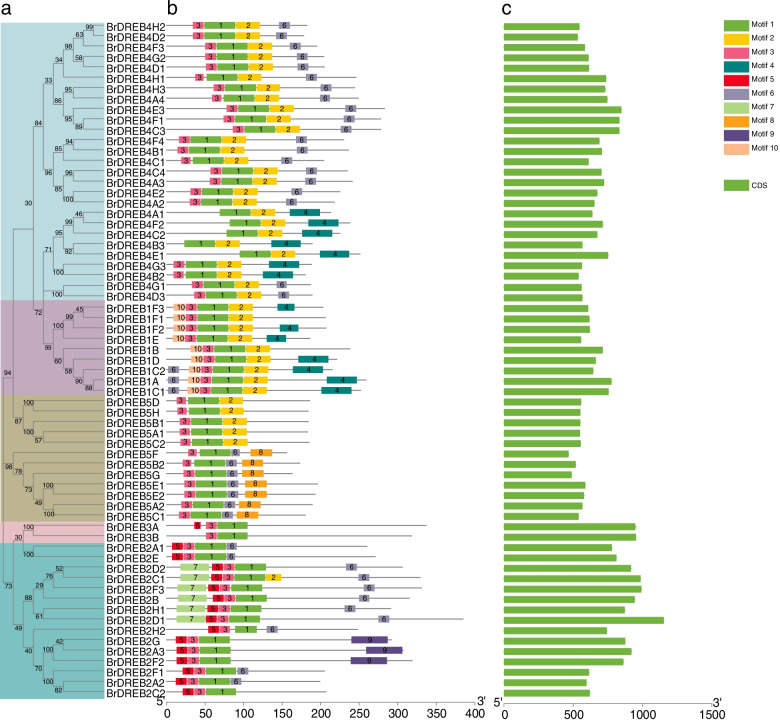
Phylogenetic tree, motif analysis, and exon-intron structure of *BrDREBs*. **a** Phylogenetic analysis of BrDREB proteins in *Brassica rapa*. **b** Conserved motif analysis of BrDREB proteins based on MEME tools. Conserved motifs are shown in different colored boxes. **c** Gene structure analysis of *BrDREBs*. Exons are indicated in green boxes

To further study the structural characteristics of the *BrDREB* gene family, we analyzed the gene structure and conserved motifs according to the full-length phylogenetic relationships (Fig. 1). Through cluster analysis, it was found that the closer the genetic relationship of genes, the more similar the number and position of motif distribution, suggesting functional similarity within subgroups. A total of ten conserved motifs were found in the DREB family of *Brassica rapa*. Motif 1 was found in all BrDREB proteins, while Motif 9 was found only in BrDREB2G, BrDREB2A3, and BrDREB2F2. In addition, structure analysis revealed that all *BrDREB* genes had no introns and only contained one exon, from which it can be inferred that the gene structure is highly conserved.

**Fig. 2:**
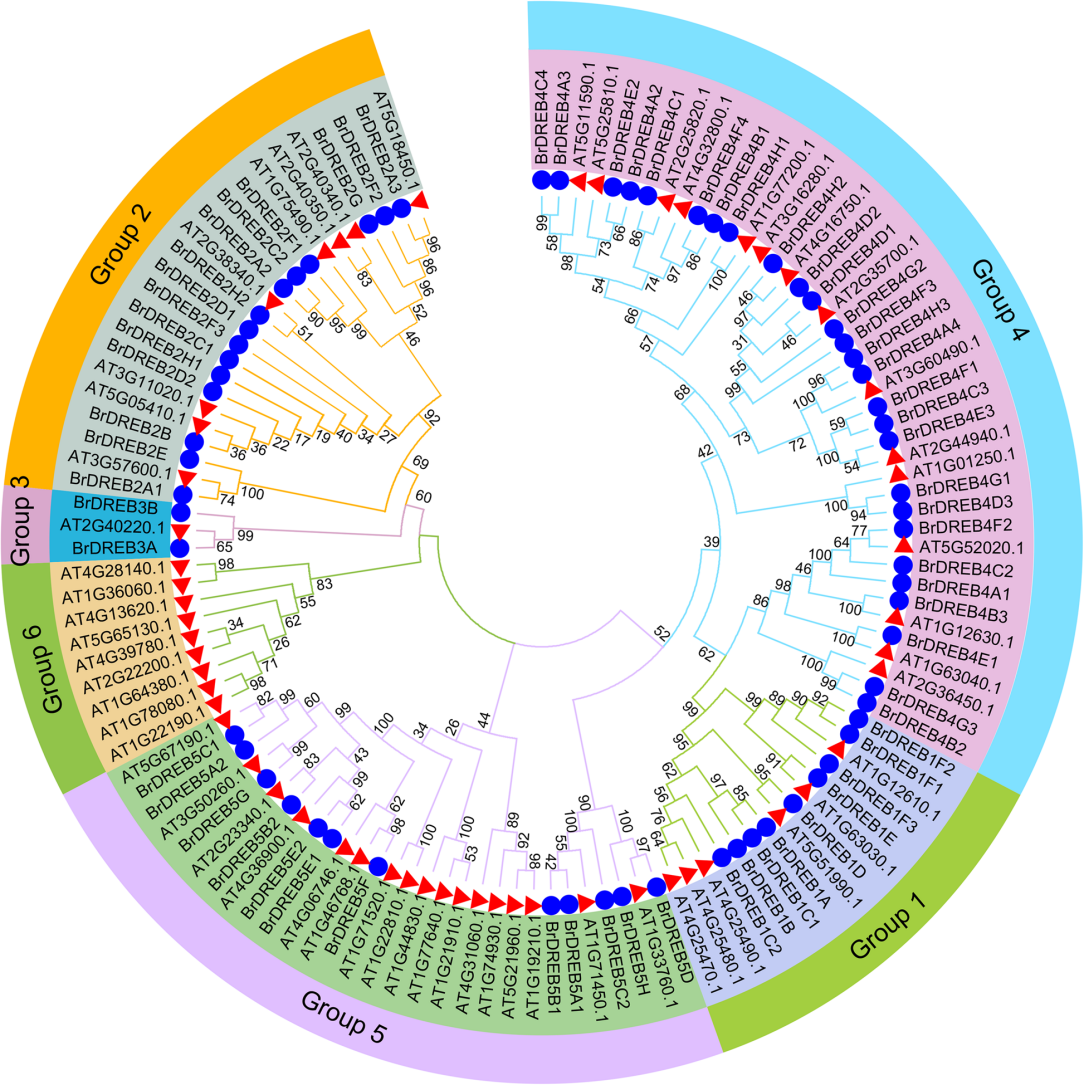
Phylogenetic tree of DREB domains from *Brassica rapa* and *Arabidopsis*. The neighbor-joining tree was constructed with DREB domains from Chinese cabbage and *Arabidopsis* using MEGA7.0 with a bootstrap of 1000

In order to understand the pedigree and functional characteristics of the *BrDREB* gene family, a multiple sequence alignment of the *DREB* family genes in Chinese cabbage and *Arabidopsis* was carried out. Thus, a phylogenetic tree was constructed (Fig. 2), and *BrDREB* family genes were divided into 6 subgroups through cluster analysis (Groups 1–6). The *BrDREB* gene was unevenly distributed among 5 subgroups. Among them, Group 4 had the most *BrDREB* genes, Group 3 had only two *BrDREB* genes, and Group 6 had no *BrDREB* genes. Through cluster analysis, it was found that the *DREB* genes of Chinese cabbage and *Arabidopsis* have high homology. It was speculated that the orthologous genes may have the same function.

### Analysis of *cis*-acting elements of the *BrDREB* gene family

**Fig. 3:**
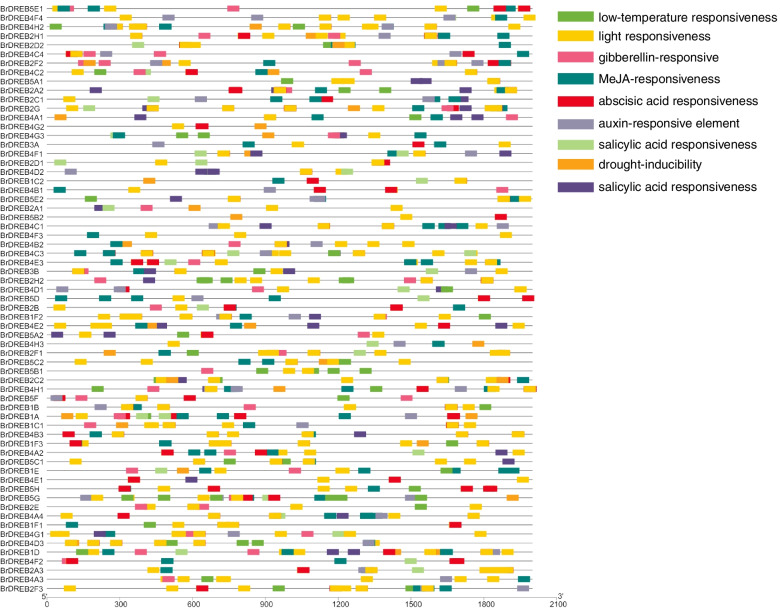
The *cis*-acting element analysis of the putative promoter of 65 *BrDREB* genes. The distribution of *cis*-elements in the 2000-bp upstream promoter regions of *BrDREBs* related to abiotic stress responses is depicted. Different *cis*-elements are represented by different colors

To further investigate the function of *BrDREB* genes, we predicted the *cis*-acting element of the putative promoter region of *BrDREB* genes using the PlantCARE database. The *cis*-acting element analysis was carried out on the promoters of the *DREB* gene family in *Brassica rapa* (Fig. 3). As shown in Fig. 3, we identified nine *cis*-acting elements according to their functional annotations, of which five elements were related to hormone response and three elements were related to adversity stress. Some diverse distribution patterns of *cis*-acting elements were observed in the promoter region of *BrDREB* genes, indicating that *BrDREB* was particular to various different biological processes. It was notable that all *BrDREB* genes contained the *cis*-acting related to hormone regulation, such as gibberellin, methyl jasmonate (MeJA), abscisic acid, auxin, and salicylic acid responsiveness elements. These results suggested that the expression of *DREB* genes in *Brassica rapa* is regulated by *cis*-elements associated with hormone signal transduction and abiotic stress tolerance.

### Chromosome distribution and collinearity analysis of the *BrDREB* gene family

**Fig. 4:**
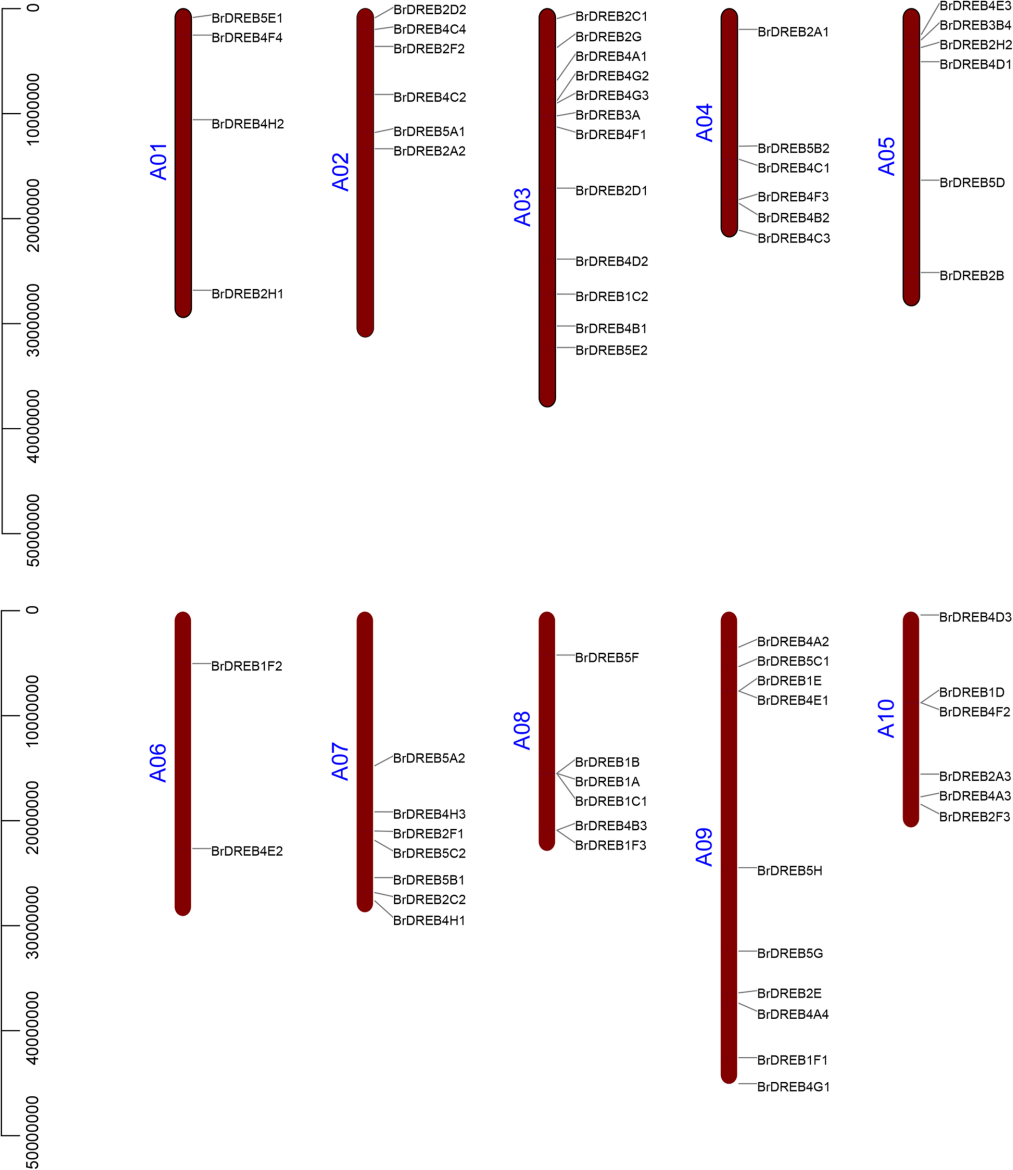
Physical locations of *BrDREB* genes in *Brassica rapa*. Chromosome distributions of *BrDREBs* are indicated based on the physical position of each gene. A total of 65 *BrDREB* genes were mapped onto the 10 chromosomes of *Brassica rapa;* the number of chromosomes is labelled on the left side of each chromosome

We analyzed the distribution of the *DREB* family genes on chromosomes, and the results showed that *BrDREB* the gene family members were unevenly distributed on 10 chromosomes (Fig. 4). Among them, chromosome A03 was the most populated, with 12 genes, while the A06 chromosome was the least populated, with only 2 genes. In addition, some *DREB* genes had tandem duplications, resulting in the presence of homologous genes in *BrDREB*, which enlarged the *DREB* genome of *Brassica rapa*.

**Fig. 5:**
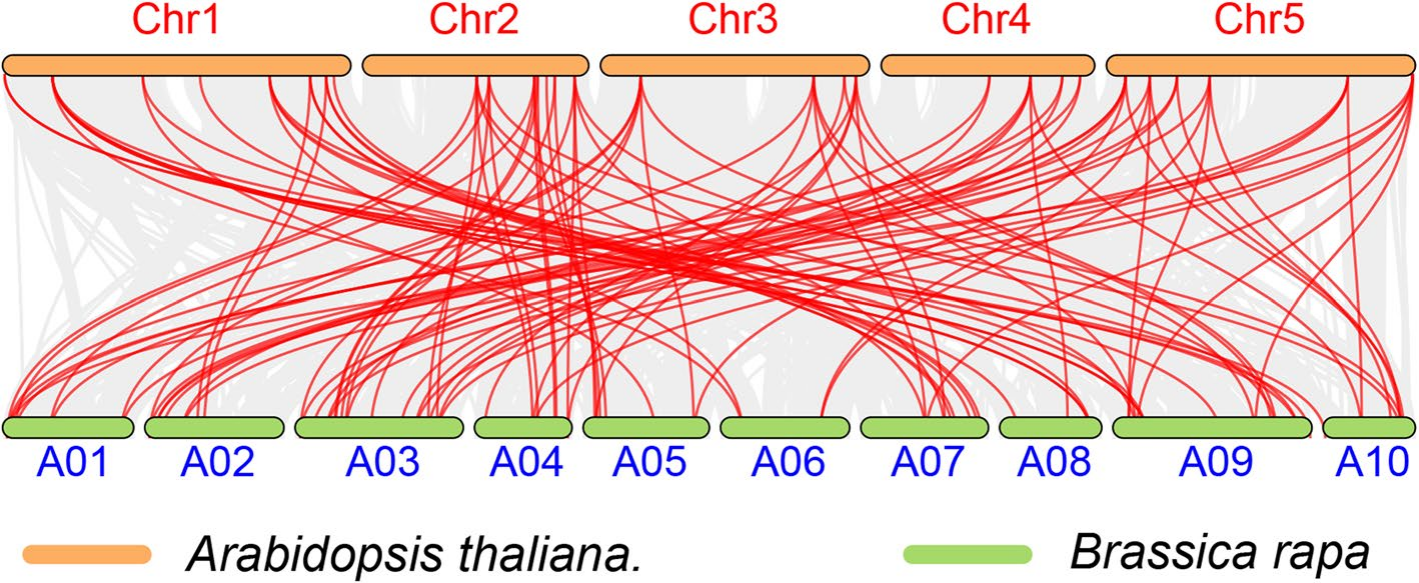
Collinearity analysis of *DREB* genes in *Brassica rapa* and *Arabidopsis thaliana*. Chromosomes of *Brassica rapa* and *Arabidopsis thaliana* are represented by orange and green bars, respectively; the chromosome label is next to the corresponding chromosome. The red curve indicates *DREB* genes with collinearity

Collinearity analysis of *DREB* genes between *Brassica rapa* and *Arabidopsis* found that a large number of members in the *DREB* gene family had a collinear relationship: 39 *Arabidopsis DREB* genes and 65 *DREB* genes (Fig. 5). There were 152 gene pairs, indicating high homology between *AtDREBs* and *BrDREBs* (Tab. S2). The ratio of nonsynonymous substitution rate (*Ka*) to synonymous substitution rate (*Ks*) of all the 152 gene pairs were below 1. These genes underwent strong purification and selection in evolution and have relatively consistent functions and effects.

### Genetic transformation of *Arabidopsis*

**Fig. 6:**
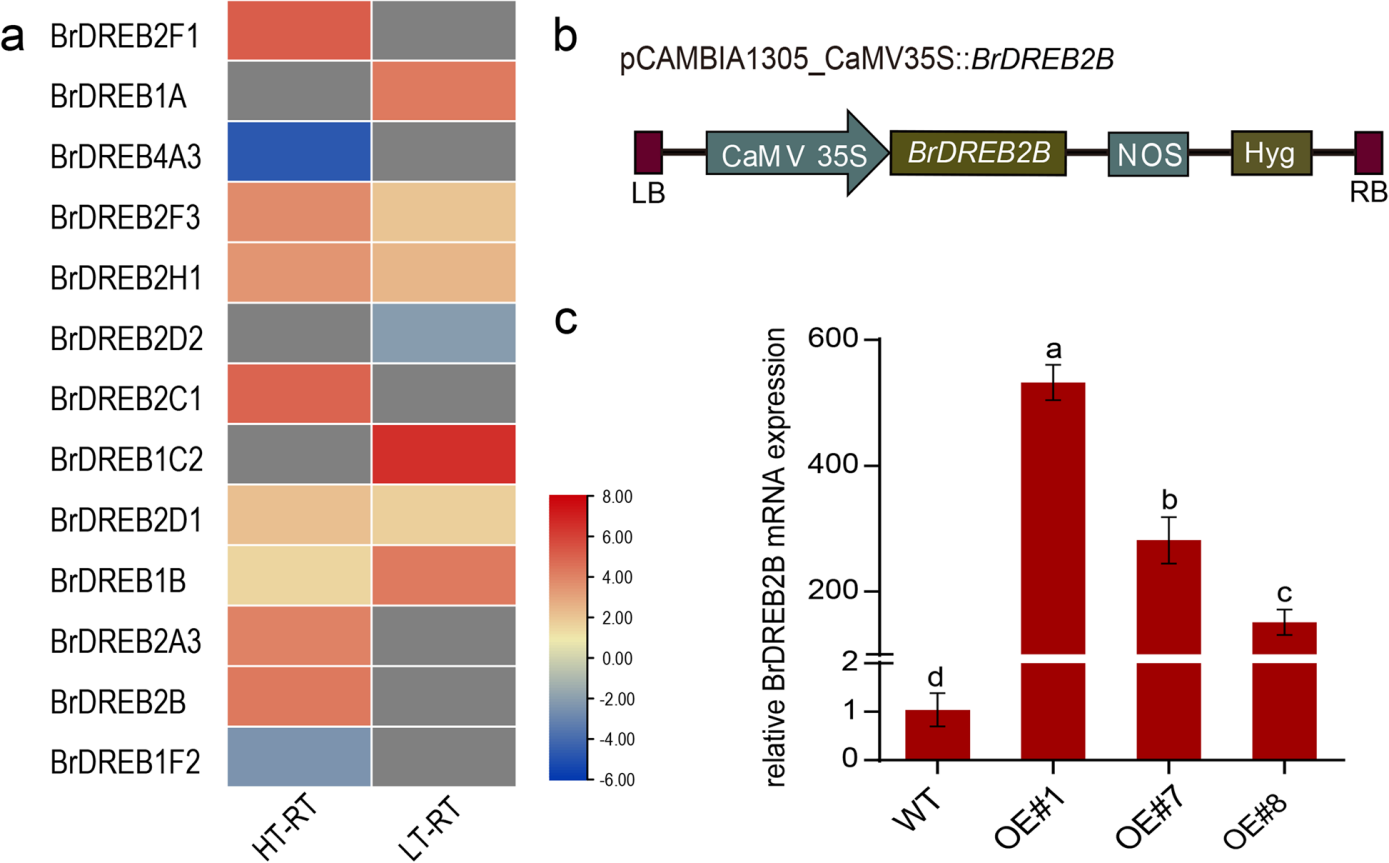
Relative expression levels of *BrDREB*s and genetic transformation of *Arabidopsis*. **a** Responses of *BrDREB2B* to adverse environmental stresses. Expression profiles under heat and cold stress were analysed. The coloured scale varies from blue to red, which indicates the low or high expression of each gene. **b** Schematic diagram of the pCambia1305-35 S-*BrDREB2B*-nFLAG-cMYC fusion protein construct. **c** Relative expression of *BrDREB2B* in T_3_ transgenic plants. WT: Col-0; OE#1, OE#7, OE#8. T_3_ plants with *BrDREB2B* on the *AtCol-0* background

To further study the function of *DREB* genes, we analyzed the expression level of *DREB* genes under heat and cold stress (Fig. 6a). We found that the expression level of the *BrDREB2B* gene increased under heat stress, so we generated transgenic *Arabidopsis* plants overexpressing *BrDREB2B* under the control of the CaMV35S promoter (Fig. 6b). Quantitative RT-PCR results showed that the transcript level of *BrDREB2B* was highly induced in selected transgenic plants, confirming that the plants were successfully transformed with pCambia1305-35 S-*BrDREB2B*-nFLAG-cMYC (Fig. 6c).

### Effects of different stresses on the germination of transgenic plants

**Fig. 7:**
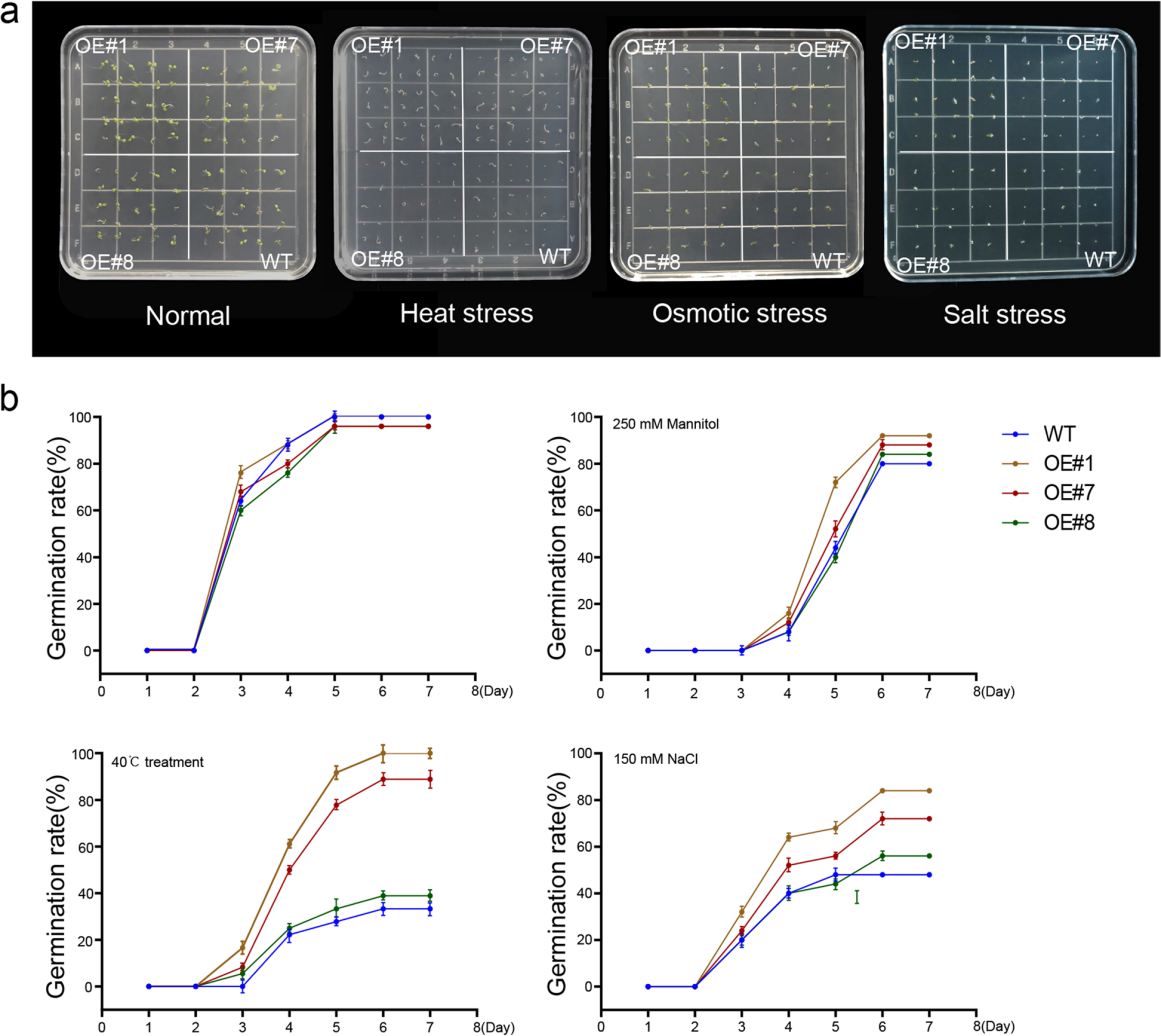
Overexpression of *BrDREB2B* increased the heat, osmotic and salt tolerance in *Arabidopsis*. **a** Phenotypes of the germination of wild-type (WT) and *BrDREB2B* transgenic lines (OE#1, OE#7, and OE#8). **b** Germination rate of wild-type (WT) and *BrDREB2B* transgenic lines (OE#1, OE#7, and OE#8). Data were quantified using three biological replicates of each cultivar. Each data point represents the mean (± SD) of three separate experiments (*p* < 0.05)

The seeds of the T_3_ *BrDREB2B* overexpression lines (OE#1, OE#7 and OE#8) and wild-type were subjected to high temperature (40 °C), salt (NaCl, 150 mM), and drought (mannitol, 250 mM) stress treatments. The germination status was observed (Fig. 7a). In the germination stage, seed germination of the wild-type and transgenic plants did not differ under normal conditions (Fig. 7). However, in the 150 mM mannitol treatment, the seed of transgenic lines displayed a significantly higher germination rate than that of the wild type. In the 150 mM NaCl and 40 °C heat stress treatments, seed germination of the wild type was seriously inhibited. In the presence of 150 mM NaCl, 50% of the wild-type seeds germinated, whereas the germination rates of the OE#1, OE#7 and OE#8 transgenic lines were 90, 70, and 60%. At 40 °C, we found 30% of the wild-type seeds germinated, whereas the germination rates of the OE#1, OE#7, and OE#8 transgenic lines were 90, 80, and 40%, respectively.

### Effects of salt stress on the root growth of transgenic plants

**Fig. 8:**
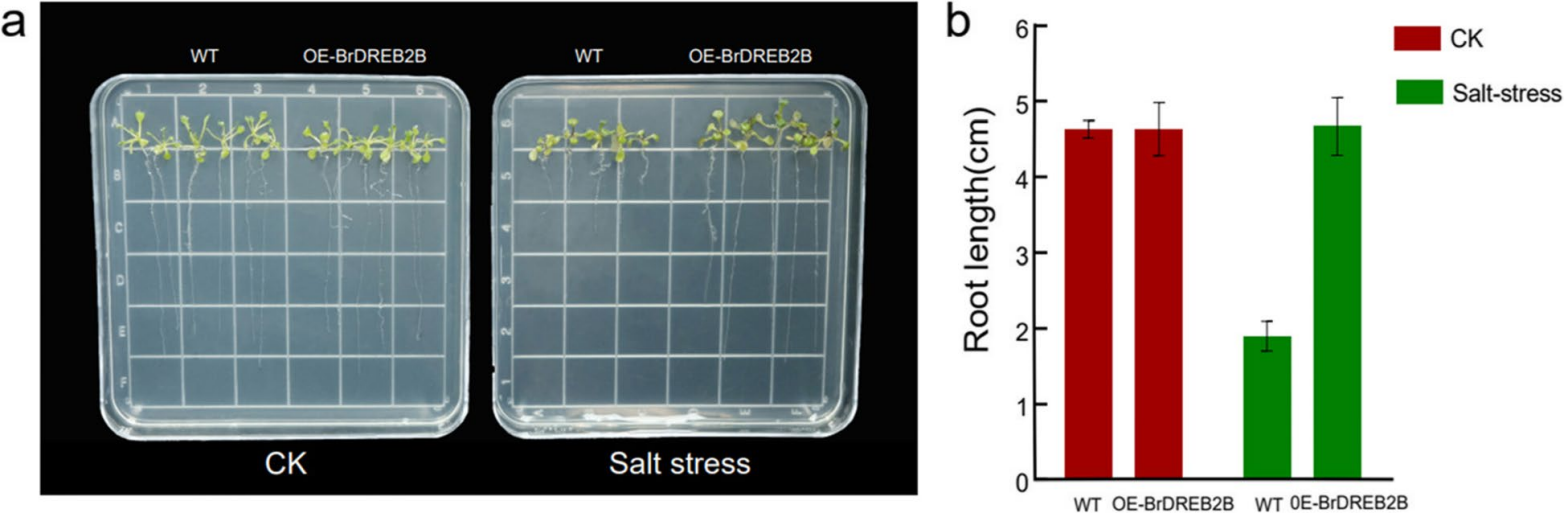
Total root lengths of transgenic *Arabidopsis* lines under mock drought stress. **a** Phenotypes of the root growth of wild-type (WT) and *BrDREB2B* transgenic lines (OE#1) under ½ MS medium with 0 mM or 150 mM NaCl for 14 d. **b** Primary root elongation of the WT and *BrDREB2B* transgenic line (OE#1) seedlings in the presence of 0 mM or 150 mM NaCl for 14 d. All experiments included three replicates. Error bars represent the mean ± standard deviation of 30 seeds

Transgenic and WT *Arabidopsis* seeds were grown on 1/2 MS medium for 5 days and then transferred to 1/2 MS medium containing 150 mM NaCl. Under the normal condition, the root length of transgenic seedlings was not significantly different from that of the wild type (Fig. 8). However, under 150 mM NaCl treatment for 14 d, the average root length of transgenic lines was 4.6 cm, which was 2.42 times that of the wild type plants. The results showed the enhanced salt tolerance of the *BrDREB2B* transgenic lines.

### Effects of different stresses on the growth of transgenic plants

**Fig. 9:**
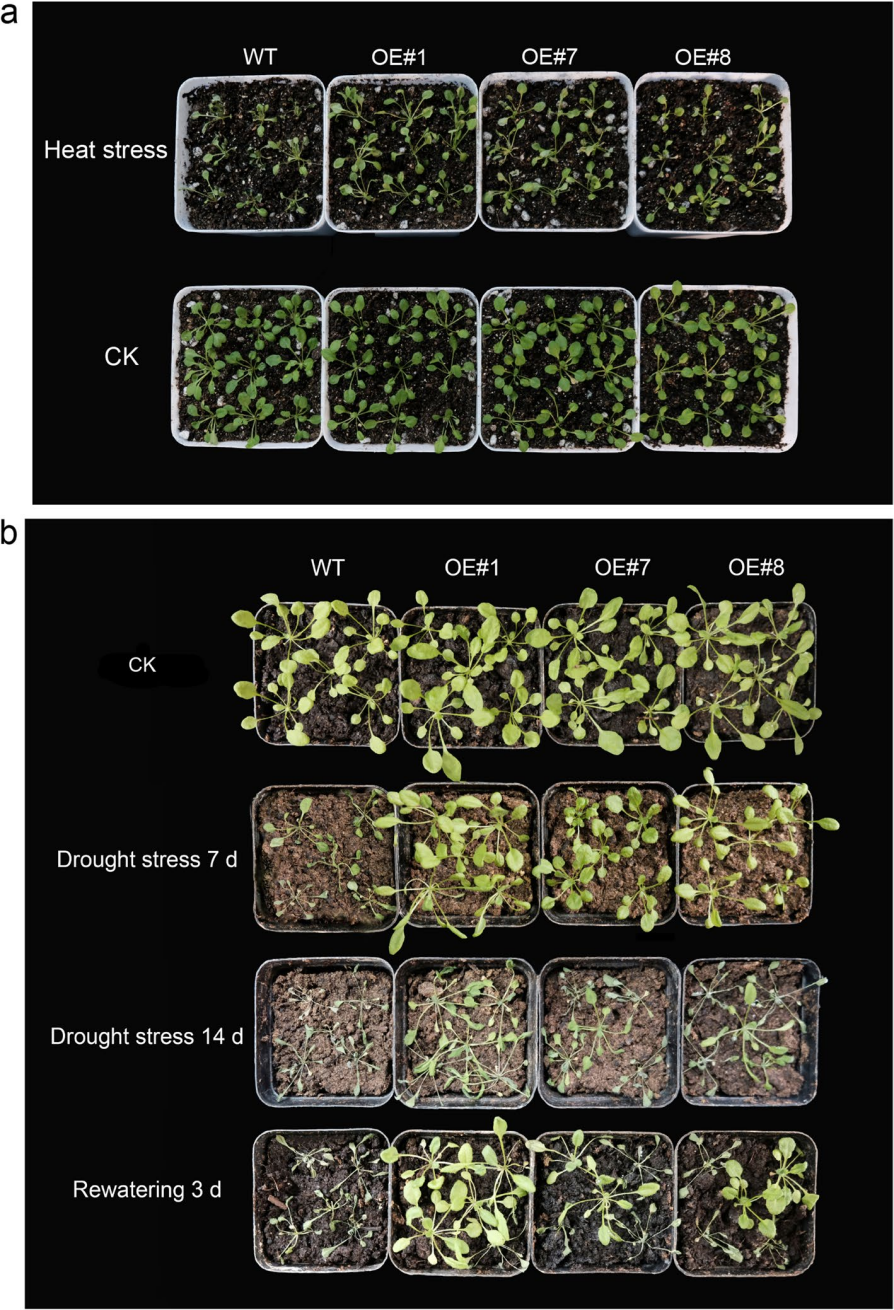
Phenotypes of transgenic *Arabidopsis* overexpressing *BrDREB2B* under heat (**a**) and drought (**b**) stresses. Each experiment was repeated three times with 16 plants

As shown in Fig. 9a, under heat stress, transgenic lines overexpressing *BrDREB2B* grew well with green leaves, while the leaves of the wild type grew weakly. Under drought stress for 7 d, some leaves of the wild-type plants were dry and curled, while leaves of the transgenic *Arabidopsis* overexpressing *BrDREB2B* were still green (Fig. 9b). Two weeks after watering was stopped, the wild-type plants showed a state of dryness and wilting, and a large number of them even died, whereas the OE#1 transgenic plants showed mild wilting, and the other two lines showed a state of leaf curling and a lack of water. After 3 days of recovery, most of the transgenic plants returned to normal growth, while the wild-type plants were weak and wilted with curled leaves. The survival rate of the OE#1 transgenic plants exceeded 90% after 3 days of rewatering, while the survival rate of wild-type plants was less than 10%, which was significantly lower than that of transgenic plants. Over-expression of *BrDREB2B* significantly enhanced the survival rate of transgenic *Arabidopsis* under heat and drought stresses.

### The effects of different stresses on the physiological indices of transgenic plants

**Fig. 10:**
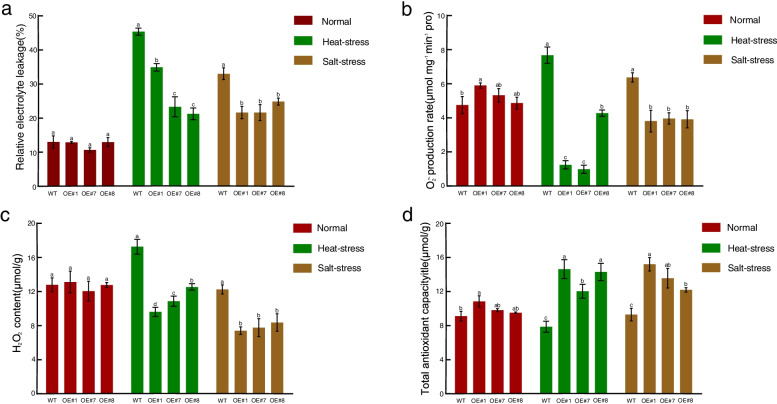
Physiological analyses of *Arabidopsis* under heat and salt stresses. The relative electrolyte leakage, O_2_^**·−**^ production rate, H_2_O_2_ content, and total antioxidant capacity in WT, OE#1, OE#7 and OE#8 plants under normal conditions (CK) and heat and salt stresses were analyzed using leaves collected after treatment. Each experiment was repeated three times, and different letters within a column indicate significant differences at *P* < 0.05

In addition, the physiological characteristics including the accumulation of H_2_O_2_ and O_2_^**·−**^, REC, and total antioxidant capacity also reflected the stress tolerance. We detected these indices in the wild type and three lines of transgenic *Arabidopsis* over-expressing *BrDREB2B* (OE#1, OE#7, and OE#8). Under normal conditions, the leaves of WT and transgenic *Arabidopsis* showed no difference. Compared with the wild-type, the REC, H_2_O_2_ content and O_2_^**·−**^ production rate of the transgenic plants under heat and salt stresses decreased, while the total antioxidant capacity increased (Fig. 10). This indicated that compared with wild-type plants, transgenic plants had greater reactive oxygen species scavenging ability, thereby maintaining cell homeostasis. These results further indicated that overexpression of *BrDREB2B* improved the tolerance of transgenic *Arabidopsis* to salt and heat stresses.

## Discussion

According to the number of AP2 domains and the existence of other DNA binding domains, AP2/ERF superfamily can be divided into AP2, ERF, DREB, RAV and Soloist subfamilies [40]. Although the sequence of AP2/ERF domain is highly conserved, each subfamily shows different DNA binding characteristics [40]. Generally, the ERF subfamily binds to an AGCCGCC sequence [41]. AP2 subfamily binds to the GCAC(A/G)N(A/T) TCCC(A/G)ANG(C/T) element [42, 43] and is regulated by microRNA (mir172) [44, 45]. The RAV family binds to the CAACA and CACCTG sequences [46]. DREB subfamily usually interacts with CCGA/CC core sequence [47].

AP2/ERF TFs have been successfully identified in many plants, such as Arabidopsis, rice [8, 48], grape [7], poplar (Populus tomentosa) [49], corn [50], wheat (Triticum wheat) [51], soybean (Glycine max) [52] and Chinese cabbage (Brassica rapa ssp. pekinensis) [36]. AP2/ERF TFs can regulate various biological processes in plant function and development [53, 54], such as drought (*SHN1*, *SHN2* and *SHN3*), salt (*AP37*, *CaRAV1*), freezing (*TaERF1*) [55–57], cell dedifferentiation (*WIND1*) [58], ABA induced crown root sprouting (*CRL5*) [59], plant height (*NSAP2*) and leaf shape [60–62].

As an important branch of AP2/ERF, DREB subfamily is used as a feasible candidate gene to improve abiotic stress tolerance of crops [40]. The DREB-type transcription factors have recently been recognized in various plants, for example, *Arabidopsis* [63], rice [8], tomato [64], tobacco [65], barley [66], sorghum [67], and maize [68]. The *DREB* genes also play a key role in plant responses to multiple abiotic stresses, including osmotic (*CkDREB*) [69], drought (*OsDREB1*) [70, 71], cold (*AtCBF1*) [72], heat (*ZmDREB2A*, *AtDREB1A*) [73, 74], and high-salt stress (*CaDREBLP1*) [75]. However, the *DREB* genes have not been systematically studied in Chinese cabbage.

In this study, a total of 65 *DREB* family members were identified in Chinese cabbage (Table S1). Combined with the previous identification of 56 *DREB* genes in *Arabidopsis*, 56 in rice, and 38 in grape, the *BrDREB* family members are more abundant. It could be due to the triploidization of the whole genome of cabbage. The duplication of chromosome fragments or the entire genome is the main source of evolution, including the generation of new gene functions and expression patterns [76]. In this study, some *DREB* genes had tandem duplications, resulting in the presence of homologous genes in *BrDREB*, which enlarged the *DREB* genome of *Brassica rapa* (Fig. 4). Furthermore, the genome size of each species also has an impact on the number of *DREB* family members. A large number of members in the *DREB* gene family had a collinear relationship between *AtDREB*s and *BrDREB*s (Fig. 5, Tab. S2). And the ratio of *Ka* to *Ks* of all the 152 gene pairs were below 1. These genes had high homology and underwent strong purification and selection in evolution and have relatively consistent functions and effects.

To investigate the evolutionary relatedness of the identified sequences together with *DREB* genes encoded by the other fully sequenced *B. rapa* and Arabidopsis, we performed phylogenetic reconstruction using the conserved *DREB* transcription factor domain. Based on phylogenetic tree analysis from *(A) thaliana* and *(B) rapa*, *BrDREBs* are classified into 6 subgroups, which is coherent with prior statements [18, 77]. However, the *BrDREB* gene was not present in group 6, so it can be assumed that a loss event occurred in the genetic evolution of *B. rapa*. The gene structure analysis revealed that *BrDREBs* contain no introns and only one exon, which is consistent with previous results for *Arabidopsis* [18]. To characterize the distribution of genes, we found that these genes are unevenly distributed on 10 chromosomes, and some genes exhibit tandem duplication. Some gene duplication events of CBF/DREB family TFs in B. oleracea were found [40]. This indicates that *BrDREB* could be generated through chromosome duplication and gene duplication events.

Furthermore, we analyzed the *cis*-acting elements on the promoters of the *BrDREB* gene. These elements are mainly related to hormone regulation (such as response to abscisic acid, salicylic acid, and auxin) and abiotic stress (such as response to drought and low-temperature stress). The *cis*-acting elements analysis predicts that the function of the *BrDREB*s could play a role in hormonal response and abiotic stress responses, which is similar to the results of the study of *MnDREB*s in mulberry [78]. Mizoi et al. found DREB transcription factors can regulate the expression of stress resistance genes through ABA-dependent and ABA-independent pathways [79]. In tomato, the ectopic expression of *SlDREB3* can increase the growth of plant roots and improve the photosynthetic rate by reducing the level of ABA [80]. Taken together, the *BrDREB*s may be involved in the regulation of hormone metabolism pathways to improve plant resistance to environmental stresses such as drought, low temperature, and salinization.

In order to further explore the role of *BrDREB2B* in abiotic stress, we constructed a pCambia1305-35 S-*BrDREB2B*-nFLAG-cMYC overexpression vector and obtained transgenic *Arabidopsis* plants. The results indicate that transgenic plants overexpressing *BrDREB2B* showed better growth status and higher survival rates after drought, high-salt and high-temperature stresses than wild-type plants. Similarly, overexpression of *OsDREB2A* [81], *TaDREB3* [12], and *GmDREB2A* [82] in *Arabidopsis* enhanced tolerance to abiotic stress. *SsDREB2D* and *SsDREB2F* play a key role in sugarcane dehydration reaction, which helps sugarcane plants recover from drought stress and low temperature stress [83]. *OsDREB2A* and *OsDREB2B* were reported to involve in the embryo and endosperm development in rice [84]. Therefore, *BrDREB2B* may play a key role in response and resistance to abiotic stress in wucai and can be used as a candidate gene for molecular breeding to increase the yield of wucai.

The accumulation of reactive oxygen species (ROS) is closely related to the environmental stress of plants, leading to oxidative damage and lipid peroxidation of plant cell membranes [85]. In this study, the relative electrolytic leakage content, H_2_O_2_ content, and superoxide anion production rate of the three overexpression transgenic lines after different stress treatments decreased. At the same time, the total antioxidant capacity increased compared with the wild-type. These results verify that *BrDREB2B* improves its tolerance to abiotic stress by maintaining the homeostasis of intracellular reactive oxygen species, which is consistent with a report for soybean [86]. In carrot, *DcDREB1A* overexpression in transgenic plants resulted in increased superoxide dismutase (SOD) and peroxidase (POD) activities under drought stress [87].

## Conclusion

In this study, 65 *BrDREBs* were identified in *Brassica rapa* and the gene structure, protein conserved domains, evolutionary relationship, and *cis*-acting elements were analyzed. There is high homology in the *DREB* gene of Chinese cabbage and *Arabidopsis*. In addition, many abiotic stress response *cis*-elements were found in the promoter regions of *BrDREB* genes. Finally, the heterologous expression of *BrDREB2B* improved the salt, heat, and drought stress tolerance of transgenic *Arabidopsis*. This work has provided a solid foundation for the investigation of the function and the mechanism of response to abiotic stress of the DREB TF family in wucai.

## Supplementary Information

**Additional file 1.**

**Additional file 2.**

**Additional file 3.**

## Data Availability

The raw RNA-Seq data used in this study have been deposited in the Nation Center for Biotechnology Information (NCBI) Sequence Read Archive (SRA) database under the accession number PRJNA694542 (https://www.ncbi.nlm.nih.gov/bioproject/PRJNA694542).
